# An Open-Road Driving Performance Task to Examine Long-Term Medical Marijuana Use and Prescription Opioid Positivity Among Adults Aged 50 Years and Older: Protocol for an Observational Trial

**DOI:** 10.2196/77944

**Published:** 2025-11-28

**Authors:** Nicole Ennis, Sherrilene Classen

**Affiliations:** 1 Department of Behavioral Sciences and Social Medicine College of Medicine Florida State University Tallahassee, FL United States; 2 Department of Occupational Therapy College of Public Health and Health Professions University of Florida Gainesville, FL United States

**Keywords:** chronic pain, driving, medical marijuana, older adults, opioids

## Abstract

**Background:**

Driving performance involves multiple underlying components of psychomotor functioning, such as attention, executive functions, and vehicle control. While the effects of acute medical marijuana and prescription opioid intoxication are known, how long-term use of medical marijuana under real-world conditions affects driving performance is unknown. Additionally, there are numerous ongoing physical and cognitive changes that affect driving performance with age. Given the proliferation of medical marijuana and prescription opioid use in adults aged 50 years and older, the prevalence of polypharmacy, and declining functional abilities, it is imperative to understand the long-term effects of daily medical marijuana use. Further, we need to understand how co-occurring use of medical marijuana and prescription opioids, in the presence of comorbidities such as chronic pain, affects real-world driving outcomes.

**Objective:**

This study aims to document the observational trial protocol. The primary goal of this study is to identify the effects of daily long-term (ie, use for >12 months daily or most days of the week) medical marijuana use on driving performance outcomes using an open-road driving performance task under real-world conditions in adults aged 50 years and older who endorse chronic or severe nonmalignant pain and to examine the combined effect of daily long-term medical marijuana use and prescription opioid use on driving outcomes. A secondary goal is to qualitatively explore self-regulation of medical marijuana and prescription opioid use in this population.

**Methods:**

We plan to test medical marijuana use as the exposure variable in adults aged 50 years and older on an open-road driving task performance as the primary outcome. The study will detail tetrahydrocannabinol exposure through ecological momentary assessment and urinalysis and will compare performance with a race-sex–matched group of non–marijuana users.

**Results:**

This study is funded by a grant from the National Institute on Drug Abuse (5R01DA057965). Recruitment began on May 19, 2025. As of November 2025, a total of 30 participants had been enrolled. Recruitment is anticipated to be completed by 2029. Publication of the complete results and data from this study is expected by 2030.

**Conclusions:**

Data from this study will identify the effects of long-term medical marijuana use and the combined effect of that use with prescription opioids to develop risk screening protocols and intervention targets for this population. The development and dissemination of screening and intervention guidelines will be the next step in this work.

**Trial Registration:**

ClinicalTrials.gov NCT06995937; https://www.clinicaltrials.gov/study/NCT06995937

**International Registered Report Identifier (IRRID):**

DERR1-10.2196/77944

## Introduction

With increasing marijuana use, cannabinoids have become one of the most frequently encountered psychoactive substances in the blood or oral fluids of drivers who are drug-impaired or involved in motor vehicle accidents [1]. The 2015 US National Roadside Survey showed that the rate of tetrahydrocannabinol (THC)–positive drivers increased 48% among all drivers [2]. Further, THC is associated with a 50% increased risk for traffic crashes [3,4], and the risk of motor vehicle collision while driving under the influence of marijuana is twice as high as when driving unimpaired [1,5]. Opioid use is associated with a 47% increased risk of crash involvement [6,7]. Further, motor vehicle collisions are among the leading causes of unintentional morbidity and mortality in North America [8,9]. While the cause of motor vehicle collisions is multifaceted, a potential contributor is the use of psychoactive medications such as prescription opioids [6,10,11]. Due to developmental life stage, older adults have a higher risk for multiple drug use, including polypharmacy [12], which puts them at a higher risk for drug-drug interactions [13]. Driving performance involves multiple underlying components of psychomotor functioning, such as attention, executive functions, and vehicle control [14]. While the effects of acute medical marijuana and prescription opioid intoxication are known [13,15-17], how long-term use of medical marijuana affects driving performance—a complex skill needed for continued independence with age—is unknown. Additionally, there are numerous ongoing physical and cognitive changes that affect driving performance with age [18,19]. Given the proliferation of medical marijuana use and prescription opioid use in adults aged 50 years and older [20-23], the prevalence of polypharmacy, and declining functional abilities, it is imperative that we understand the long-term effects of daily medical marijuana use. Further, we need to understand how co-occurring use of medical marijuana and prescription opioids, in the presence of comorbidities such as chronic pain, affects real-world driving outcomes.

Findings regarding the effects of long-term marijuana use are mixed. Some evidence indicates that long-term, heavy cannabis use (ie, daily or almost daily use) may cause impairment in cognitive and motor functions [24]. Long-term heavy use is associated with decreased motor control, decreased attentional control in both sustained and divided attention, greater cognitive impairment, and greater short-term memory deficits [24,25]. Current heavy use is also associated with decreased vehicle control [1]. However, some studies suggest that impairment due to long-term heavy use does not persist after extended abstinence periods and that mild to moderate use is not associated with impairment [26].

For mid- to late-life adults, participation in driving activities allows them to meet their social roles and stay independent [14,27,28]. Factors such as age-related functional impairments due to chronic pain and side effects of medications such as medical marijuana and prescription opioids can compromise participation in driving and lead to heightened crash risk [28-30]. In the United States in 2015, more than 85% of adults aged 65 to 84 years and nearly 70% of adults aged 85 years and older were licensed to drive [31]. By 2030, the number of older adults is projected to account for 21% of the US population, and most older adults will retain their driver’s licenses [32,33].

To date, no studies have systematically assessed driving performance in a rigorous and ecologically valid manner accounting for long-term medical marijuana use and the combined effect of prescription opioid use in adults aged 50 years and older, who endorse chronic or severe nonmalignant pain. Further, studies examining how older adults self-regulate prescription medication use and driving behavior are limited, with none rigorously examining medical marijuana.

Thus, the overall objective of this study is to address the research questions: Does daily long-term medical marijuana use affect performance on an open-road driving task in adults aged 50 years and older who endorse chronic or severe nonmalignant pain, and what is the combined effect of long-term medical marijuana and prescription opioid use on driving performance outcomes in this population?

## Methods

### Ethical Considerations

This research was approved by the Florida State University Institutional Review Board (STUDY00005560) in December 2024. Informed consent is obtained prior to beginning any study procedures. Participation is completely voluntary. Participant privacy and data confidentiality are protected through multiple measures, including a private interview setting for all study procedures, a Health Insurance Portability and Accountability Act (HIPAA)–compliant data storage platform (Research Electronic Data Capture [REDCap; Vanderbilt University]) and text message delivery application (Mosio), and coding of all data so that it cannot be associated with any individual. Participants will receive up to US $245 if all assessments are completed as directed in the study protocol.

### Study Design

**Figure 1:**
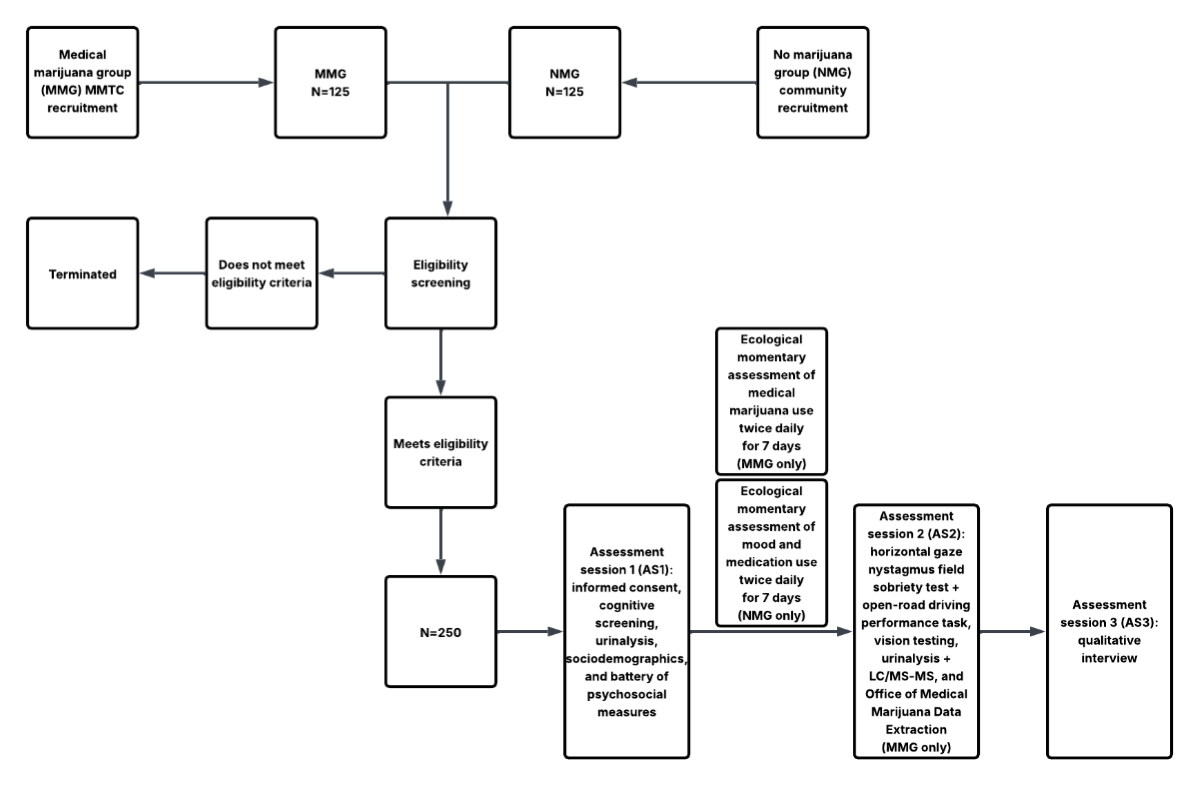
Study flowchart. LC/MS-MS: liquid chromatography–tandem mass spectrometry; MMTC: Medical Marijuana Treatment Clinics of Florida.

We are using a two-group observational study design with a group with medical marijuana use for more than 12 months daily or most days of the week (medical marijuana group [MMG]) versus a race-sex–matched group with no marijuana exposure in the past 10 years and no history of daily use (no marijuana group [NMG]). We chose 10 years as the no–marijuana-exposure window to ensure that recreational use was not a confounder. We will test the difference between long-term medical marijuana exposure and no marijuana exposure on driving performance outcomes in adults aged 50 years and older who endorse chronic or severe nonmalignant pain through an open-road driving assessment. There will be up to 3 in-person assessment sessions over approximately 10 days. Assessment session 1 (AS1; the first assessment session) is the onboarding session, which will include informed consent, sociodemographics, a battery of psychosocial measures, and urinalysis to test for the presence of marijuana, opioids, and other commonly misused substances. After AS1, we will use ecological momentary assessment to capture medical marijuana use (MMG) or mood and medication use (NMG). MMG participants will complete twice-daily assessments of medical marijuana use for 7 days. To attention match, the NMG will complete twice-daily assessments of mood and any medication use daily for 7 days. Assessment session 2 (AS2; the second assessment session), the open-road driving performance task, will occur approximately 8 days after AS1 and, for MMG participants, 8-12 hours after the last medical marijuana use. At AS2, vision screening to ensure appropriate visual acuity and urinalysis will be completed before the drive. Additionally, we will request permission from MMG participants only to extract Office of Medical Marijuana Use (OMMU) purchase history data, which include the dispensary name, date of purchase, quantity of medical marijuana purchased, and route of administration. Assessment session 3 (AS3; the third and final assessment session) will be a qualitative interview with a subset of participants (n=60) regarding self-regulation of medical marijuana and prescription medication use, to be completed no later than 3 days after the drive (Figure 1).

This study is designed to address an urgent public health issue: “What are the effects of chronic, habitual marijuana use on adults aged 50 years and older?” Mid- to late life is a sensitive time period that involves significant changes that can impact quality of life, and understanding how chronic habitual marijuana use affects cognitive and psychomotor function is a significant and vital public health question. This study is designed to obtain data needed for the development of guidelines and best practices for use among adults aged 50 years and older who use marijuana daily or most days. The primary outcome, driving performance, is a vital measure of continued independence with aging and represents a real-world activity that captures cognitive, motor, memory, and executive functions in a single task. Therefore, the estimand of this study is to understand the difference in driving performance errors (ie, lane maintenance errors, speed regulation errors, adjustment-to-stimuli errors, yielding errors, signaling errors, vehicle positioning errors, visual scanning errors, and gap acceptance errors) among adults aged 50 years and older who have used medical marijuana daily or most days for at least the past year (MMG), as compared race-sex–matched adults who do not consume any marijuana (NMG). Further, we are intentionally using an 8- to 12-hour timeframe, as that is consistent with how adults in this population report marijuana consumption behavior in relationship to driving. This study design maximizes both ecological and external validity by estimating group differences in performance that reflect long-term neuroadaptation and real-world use patterns rather than acute intoxication. In addition, urine metabolites are collected before each drive so chronicity of marijuana use will be on a continuous spectrum as measured by 11-nor-9-carboxy-tetrahydrocannabinol (THC-COOH) output. The focus of our study design is to better understand chronic habitual use and whether there are any impacts on driving performance under real-world conditions.

### Primary Outcome

#### Open-Road Course Driving Performance Test

Our standardized road course is a fixed route in Gainesville, Florida, with a gradual progression of driving difficulty. The scoring mechanism is based on errors, which are used to evaluate driving maneuvers. A standardized scoring sheet with a total of 49 driving maneuvers and 8 types of possible driving errors is used by the certified driver rehabilitation specialist (CDRS) or driver rehabilitation therapist (DRT) while the participant is driving. The driving errors are defined in the subsequent subsections.

#### Lane Maintenance Errors

These refer to the lateral positioning of the vehicle during driving maneuvers such as turns, straight driving, lane changes, and while stopped, reflecting the ability to maintain steering control.

#### Speed Regulation Errors

These reflect the ability to follow and maintain speed limits and adequately control acceleration and braking features of the vehicle; examples include not coming to a complete stop at a stop sign, traveling too slowly or too fast, inadequate merging speed, and abrupt or inappropriate braking or acceleration.

#### Adjustment-to-Stimuli Errors

These capture the ability to adjust appropriately to changing road sign information, other vehicle movements, and pedestrian movements, and to recognize potential hazards; examples include not adjusting speed for posted limits, not following proper directions given by the evaluator, choosing an improper lane from posted signage, and improper responses to traffic, pedestrian, or cyclist movement.

#### Yielding Errors

These refer to the ability to recognize common rules of road safety and to give the right-of-way when appropriate; these are assessed at 4-way or 2-way stop intersections when other vehicles are present, during right turns on red, and during merges.

#### Signaling Errors

These reflect the proper use of turn signals; examples include leaving the turn signal on, not using the turn signal when turning, using the turn signal inappropriately (eg, using the wrong signal for a given turn), or signaling for too short a duration before a maneuver.

#### Vehicle Positioning Errors

These refer to the anterior-posterior position in relation to other vehicles, objects, and pavement markings; they capture following distance during forward movement and vehicle spacing during lane changes and merges. Examples include traveling too closely or tailgating, having an inadequate space cushion during a merge or lane change, and stopping across a crosswalk or too far back from either pavement markings or other vehicles.

#### Visual Scanning Errors

These refer to demonstration of visual scanning of the driving environment; examples include not checking blind spots or mirrors during turns and lane changes and not looking left or right before proceeding through an intersection.

#### Gap Acceptance Errors

These refer to choosing an appropriately safe time and spacing distance to cross in front of oncoming traffic in the context of an unprotected left turn; these are based on evaluator judgment given the speed of oncoming traffic and the number of lanes to be crossed.

#### Scoring Criteria

Scoring in this study is based on assessing driving errors to discern how participants perform on the road and to identify driving performance deficits. The 8 driving errors are being measured on a scale from 0 (physical intervention indicating very poor driving performance) to 3 (no driving errors indicating good driving performance). For each maneuver, a maximum score of 3 is given if no errors are committed. Specifically, the scoring is as follows: 3 indicates no errors, 2 indicates any error in any driving behavior for the given maneuver, 1 indicates that the evaluator must use verbal cues or repeat instructions (not hearing-related) to modify or change driving behavior, and 0 indicates that physical intervention (such as grabbing the steering wheel or using the auxiliary brake) is required. At the end of the driving course, the sum of the points attained is computed as the sum of maneuvers score (SMS), ranging from 0 to 147, with 147 indicating a perfect driving score (zero errors) [34,35], which provides detailed information to identify deficits in the participants’ driving performance.

### Study Setting and Recruitment

Participants will be recruited from Medical Marijuana Treatment Clinics of Florida (MMTC) in Gainesville, Ocala, and The Villages. To date, at the 3 locations from which we will recruit, medical marijuana physician services are provided to 4653 adults aged 50 years and older, with 3119 having at least 1 recertification appointment or more. Recertification in the state of Florida occurs every 210 days; therefore, we have an adequate number of potential participants to meet the sample size demands for the MMG. NMG participants will be race-sex–matched adults aged 50 years and older who endorse chronic or severe pain, recruited from the community using flyers and handouts, newspaper advertisements, internet advertisements, social media, in-person lectures and events, and contact by mail, email, and through the infrastructure and support of Oak Hammock at the University of Florida (UF), a residential retirement community.

### Retention

First, we will develop a strong collaborative relationship between participants and members of the research team. Second, participants welcome participation when they know that they are contributing and that their efforts have the potential to improve other people’s experiences. Therefore, we will send information on pain management, other related resources, and local events that highlight the benefits of research. Finally, to enhance retention for this proposal, we will obtain additional contact information, communication preferences, and use dedicated research staff time for connecting, tracking, and follow-up.

### Sample Size and Power

Power estimates for this study are based on the number needed for the subanalysis to ensure we have sufficient power to answer aim 2. Using G*Power for the *F* test, we calculated the sample size for a small effect size (*f*=0.20) based on the expectation of a smaller effect size for subgroup analyses than for the main analyses [36]. Therefore, with an α of 0.5 and power set at 80%, a total sample size of 198 is needed to detect a small effect between subgroups of medical marijuana only, medical marijuana and prescription opioids, prescription opioids only, and no use at all. However, to ensure we have the full sample needed, we are planning for a potential early termination rate (eg, drive not completed due to intoxication or other reasons) of 5%, which indicates we need a sample size of 250—as 198 + 10 (5%) = 250 (125 per group). We anticipate a medium effect size for aim 1 based on the first ROAD ACE (Reactions of Older Adults Driving After Cannabis Exposure) project (1R21DA048067) that examined driving errors and medical marijuana 7-day pattern of use in a driving simulator. We found a Pearson *r*=0.459, which is equal to a medium effect size (see preliminary data above). Therefore, we will have sufficient power to test the effects in aims 1 and 2.

### Participants

Participants will include 250 adults aged 50 years and older who have been registered with the state of Florida for at least 12 months to obtain medical marijuana and report use daily or most days of the week (n=125), as well as race-sex–matched participants with no marijuana use in the past 10 years and no history of daily use (n=125). Textbox 1 provides the inclusion and exclusion criteria. To examine age as an independent factor, we will ensure that age variability is balanced between the two groups. We selected a 10-year no marijuana exposure window to ensure that recreational use does not confound our findings. We chose to match participants by sex to ensure that sex differences do not account for driving performance outcomes. We also chose to match participants by race to ensure that differences in psychomotor functioning by race among older adults do not account for the findings. Groups will be balanced by age (±1 year) to allow for observation of group differences by age.

**Inclusion and exclusion criteria.**

**Inclusion criteria**

- Aged 50 years and older who endorse chronic or severe nonmalignant pain
- Resident of Florida with an active and valid Florida driver’s license
- Active medical marijuana card for >12 months issued by the state of Florida
- Can communicate in English
- Willing and able to complete study procedures
- Race-sex match who has not used marijuana in the past 10 years with no history of daily use

**Exclusion criteria**

- Active psychosis
- Lost or revoked driving privileges
- Score below 24 (out of 30) on the Montreal Cognitive Assessment (MoCA)
- Control participant who tests positive for marijuana use by urine test or reports any marijuana use in the past 10 years

### Study Procedures

#### Screening

Participants referred to the study by MMTC, community-dwelling adults who self-nominate for participation, or those identified by our recruitment specialist will undergo preliminary screening for eligibility, including questions related to chronic or severe pain, frequency of medical marijuana use, date of last marijuana use, Florida medical marijuana card status, and Florida driver’s license status, as well as basic sociodemographic information. Once eligibility has been verified, participants will be scheduled for their first assessment session. They will be instructed to bring a valid Florida driver’s license, a valid Florida medical marijuana card (MMG only), and a list of all current prescription medications, over-the-counter medications, vitamins, and supplements with the names, doses, and frequency of administration for each. Screening IDs will be separate from participant IDs, which will be assigned after study enrollment and informed consent. Screening data will be used for the Consolidated Standards of Reporting Trials (CONSORT). Figure 1 provides the study flow.

#### Assessment Session 1 (Onboarding, Informed Consent, and Questionnaires)

At the AS1 study appointment, participants will be greeted by a study research assistant who will introduce the study and provide informed consent. Following informed consent, participants will be screened for cognitive fitness to proceed using the Mini-Mental State Examination–Second Edition Brief Version (MMSE-2:BV), a 16-item screener used to efficiently assess cognitive function [37], to ensure that participants are eligible for the study. If a participant scores fewer than 14 points on the MMSE-2:BV, the study team will administer the Montreal Cognitive Assessment (MoCA), a 30-item screener emphasizing attention and executive function that is used to detect cognitive impairment [38]. Participants must score at least 24 points on the MoCA to proceed. Participants will then complete an assessment battery that includes sociodemographic data and psychosocial measures, including detailed assessments of medical marijuana and other substance use, pain levels, and overall current health status. We will obtain the past 3-year driving record from the Division of Motor Vehicles using the current ROAD ACE protocol. Participants will be compensated US $50 for this assessment.

#### Assessment of Daily Use

After the AS1 study visit, participants will be instructed to track their medical marijuana or mood and medication use for 7 days at home. Participants will be prompted by Mosio, a HIPAA-compliant text messaging service supported by REDCap, to respond to use surveys twice per day by entering all medical marijuana use (MMG) and mood and medication use (NMG) for 7 days. The surveys will begin 7 days before AS2, with the final survey administered the evening before AS2. All data gathered by the Mosio platform (protected health information or non-protected health information) will be subject to the same security, privacy, and breach notification measures that apply to all entities subject to HIPAA standards, and the Mosio platform is bound by the business agreement established with Florida State University. Mosio may also be used to communicate other study-related messages to participants and within the research team. NMG participants will be compensated in the same manner as MMG participants for completing daily assessments of their mood and medication. Participants will be compensated US $10 per day, with a US $5 bonus for providing data for all 7 days.

#### Assessment Session 2 (Driving Performance Task)

Participants will arrive at the UF Smart House either by Uber or by a driver of their choice. For MMG participants only, AS2 will take place 8-12 hours after the last medical marijuana use. Upon arrival at the Smart House, a study team member will complete vision screening with the UFOV (Posit Science) and Optec 5000 (Stereo Optical) visual analyzer machine and collect a urine sample for urinalysis. A portion of the urine provided by MMG participants will be reserved for analysis using liquid chromatography–tandem mass spectrometry, which provides a more precise measurement of the level of THC-COOH, a metabolite of THC, present in urine. We will request permission from MMG participants only to extract OMMU purchase history data, which includes the dispensary name, date of purchase, quantity of medical marijuana purchased, and route of administration.

The CDRS or DRT will begin the open-road driving performance task evaluation, which begins with a standardized field sobriety test to detect the presence or absence of horizontal gaze nystagmus, the involuntary jerking or sudden eye movement that naturally occurs when the eyes gaze to the side [39]. The evaluator will instruct participants to focus on and follow the trajectory of a slow-moving horizontal object, typically a pen, presented directly in front of their face. The evaluator will observe both the right and left eye for 3 distinct signs: lack of smooth pursuit, distinct nystagmus at the maximum eye deviation (N_max_), and onset of nystagmus prior 45° deviation (N45) [40]. These signs will be recorded as “present” or “absent,” and participants will be classified as “failed” if all factors are noted as “present.” Participants who fail the sobriety test will be informed that they cannot complete the drive and will be compensated US $20 for a partial AS2 visit.

After sobriety is ascertained, the task continues with a “warm-up” period using a dual-brake program vehicle (a 2019 Toyota Camry or 2020 Toyota Corolla) in a parking lot. The evaluator will review the general controls for mirrors, seats, and automatic transmission shift. Drivers will perform simple maneuvers and practice perpendicular or angled parking for approximately 3 minutes. The course is designed to progress from simple to moderate and then high-complexity environments. The parking lot or “off-road” area transitions directly into a residential or simple driving environment. To ensure adequate exposure within each level of difficulty, the course is designed to include at least 3 examples of each type of maneuver within each level of difficulty. Multimedia Appendix 1 contains road course documentation and scoring details. The assessments will take place during daylight hours from mid-morning through late afternoon—approximately 9 AM to 4 PM. Assessments will be cancelled if the clinical evaluator deems the conditions unsafe due to inclement weather or unsafe road conditions. The complete course consists of 49 maneuvers through Gainesville, Florida. The driving performance form is designed so a single evaluator can document observed driving errors adequately and remain attentive to the driver and surrounding conditions. The fixed route ensures all participants are given the same maneuvers. The evaluator will check observed errors from a list of behaviors under each maneuver. The SMS is the total number of points accumulated over the set number of maneuvers divided by the total possible number of points (49 maneuvers × 3 = 147 possible points, indicating perfect driving). Participants will be compensated US $50 for this assessment and an additional US $20 as a transportation supplement for expenses such as gas or rideshare fees spent traveling to the Smart House.

#### Assessment Session 3 (Qualitative interview)

Participants will be a convenience sample recruited from the MMG and NMG after AS2. We anticipate that 60 participants (30 per group) will be adequate to reach qualitative saturation [41], and interviews will continue until saturation is reached. We will ensure that groups are balanced by age, race, and sex to ensure the breadth of perspectives from both sides. Interviews will be one-on-one, semistructured, and conducted using an interview guide; sessions will be audio recorded and transcribed using a HIPAA-compliant research transcription company. The goal of this aim is to understand self-regulation of use, including types of products used, THC/cannabidiol percentages, and quantity of use, by integrating qualitative findings and quantitative data to identify markers for risk screening, use guidelines, and intervention targets for this population. Participants will be compensated US $50 for completing the qualitative interview. Participants may receive up to US $245 if all assessments are completed as directed in the study protocol.

### Study Conditions

#### Exposure: Medical Marijuana Group

Any patient who has been certified for medical marijuana use and has held an active medical marijuana card in the state of Florida for at least 12 months, is aged 50 years or older, endorses chronic or severe pain, and reports using medical marijuana purchased through the state of Florida dispensation system daily or most days of the week for the past year, as documented by purchases over the course of the year in the OMMU database, has an active Florida driver’s license and the visual acuity to perform the task with or without vision corrective apparatus (glasses or contacts). Medical marijuana was legalized in Florida in January 2017. Per Florida state statute 381.986, residents of Florida have access to marijuana for medical use if authorized by a qualified medical marijuana physician. All medical marijuana patients must register with the state for access to medical marijuana, which includes the certification process identifying a qualifying condition, the reason the patient is authorized to use medical marijuana, and tracking all purchases through the OMMU. Qualified medical marijuana patients are allowed to purchase up to 2.5 ounces of smokable flower every 35 days, and limits on the milligrams of all other routes of administration are set by the physician. Patients must be recertified to use medical marijuana every 210 days (7 months). We will track dosage, frequency, type of product used (high vs low cannabidiol/THC), and route of administration. All medical marijuana participants will be recruited from the MMTC, our medical marijuana recruitment partner. Participants will be allowed to complete the drive regardless of a negative or positive urine screen for opioid use. Our current data suggests that approximately 40% of medical marijuana patients will also test positive for opioid use.

#### No Marijuana Use Group (Past 10 Years)

NMG participants will be race-sex–matched adults aged 50 years and older who endorse chronic or severe pain, recruited from the community using flyers and handouts, newspaper advertisements, internet advertisements, social media, in-person lectures and events, and contact by mail, email, and the infrastructure and support of Oak Hammock at the UF—a residential retirement community. We will also phone individuals enrolled in the UF Clinical and Translational Science Institute’s Integrated Data Repository, a database organizing clinical information across UF Health’s clinical and research programs [42]. Individuals enrolled in UF Health services or taking part in UF research studies can provide consent to be contacted within this system, and data can be queried using National Institutes of Health’s Informatics for Integrating Biology and the Bedside tool, which provides researchers with a HIPAA-compliant and UF Institutional Review Board–approved limited dataset [43]. In querying this dataset for adults aged 55-84 years (adults aged 45-54 years were grouped together and thus excluded from the query) with who endorse chronic or severe nonmalignant pain, the tool reported 7463 potential participants who have consented to be contacted for research participation. Participants will be allowed to complete the drive regardless of a negative or positive urine screen for opioid use. Our current data suggests that approximately 25% of control participants will also test positive for opioid use, with literature estimating of up to 50% of patients with chronic or severe nonmalignant pain using opioids.

#### Data Blinding

Due to the extent of participant engagement with the CDRS and DRT, we will ensure that the CDRS and CDRT, who are responsible for the primary outcome, are blinded to participant condition to minimize any potential bias.

### Participant Data Protection

Any computers or tablets containing identifying data, such as participant logs, will be protected by multiple security codes using standards set by Florida State University. During data analysis, all identifying information, except the participant identification number, will be removed. The Intervention Research Advancing Care Excellence laboratory (director: NE) and Institute for Driving, Activity, Participation, and Technology (director: SC) benefit from outstanding security, backup, and technical support provided by the University of Florida Clinical and Translational Research Institute and the College of Public Health and Health Professions. Data are collected at the UF Smart House and are managed by our central data management team. All electronic medical records marijuana data will only be accessed by core study staff with the appropriate training and prior institutional review board approval.

### Safety Monitoring

#### Primary Outcome

The safety of all participants will be ensured through: (1) sobriety testing, (2) vision screening, (3) no drives conducted in rainy conditions, (4) all drives completed during daylight hours during and periods of lower traffic congestion, and (5) use of a dual-brake controlled vehicle equipped with an eye check mirror for the CDRS or DRT. In addition, a closed-road driving observation period will be conducted by the CDRS or DRT, during which any safety concerns will result in termination before entering the open road. The CDRS or DRT will provide verbal cues as needed and take over steering if necessary to prevent harm, as trained and in accordance with best practices.

#### Mitigation of Risk

Our on-road course is highly regulated. The CDRS or DRT will observe for safety, provide instructions to the driver, and implement interventions such as braking the vehicle or taking over steering if necessary. No drives will be conducted in moderate to heavy rainy conditions (as determined by the CDRS or DRT), and all drives will be completed during daylight hours and midday during periods of lower traffic congestion. During the AS2 drive study visit, we will check participants’ visual acuity and motor skills. After those steps, we will teach participants about the vehicle they will drive—a 2019 Toyota Camry or a 2020 Toyota Corolla dual-brake vehicle with in-vehicle information systems (eg, a back-up camera) and advanced driver assistance (eg, emergency braking control) features—and make sure they know how to operate the vehicle’s controls (eg, steering, brake, gas). None of the other smart features (eg, adaptive cruise control or lane centering system) will be activated during the drive.

### Data Analysis Plan

We will initially compute descriptive statistics to provide demographic and clinical characterization of the study sample. We will examine whether the MMG and NMG participants differ at study entry on potential confounding variables before testing study hypotheses and we will test medical and demographic variables as predictors of study outcomes. Medical and demographic variables that are significant predictors will be used as covariates and controlled for in subsequent analyses. Highly skewed covariates will be analyzed using spline, log transformation, or other acceptable normalization (eg, square root transformation). Finite mixture modeling and generalized linear modeling, accounting for individual- and group-level outcomes, will be used to test the study hypotheses. Primary study hypotheses will be tested using analysis of covariance (ANCOVA), given that the primary outcome SMS is a continuous variable (range 0-147) and inclusion of covariates will reduce error variation and increase statistical power, particularly as this study lacks random assignment.

### Hypotheses Testing

#### Hypothesis 1a and 1b (Driving Performance—Between-Group Analyses)

To answer the question of whether long-term medical marijuana use affects behavioral driving outcomes, we will compare the SMS between MMG and NMG using an independent 2-sample *t* test. ANCOVA will then be used to determine whether the two groups differ on SMS by adjusting for significant sociodemographic, driving, behavioral, and clinical variables. A model-building approach, which will include age, will be used, and analyses will be performed with and without covariate adjustment, since sample-dependent variables may explain some outcome variance. Tukey’s honestly significant difference test or Tukey-Kramer test will be used to estimate the clinical significance of the findings.

#### Hypothesis 2a (Prescription Opioids—Combined Effect)

To test the hypothesis that prescription opioid use will be associated with worse outcomes on driving performance (SMS), we will conduct a 2-sample *t* test to see if SMS differs in prescription opioid users versus nonprescription opioid users, regardless of medical marijuana use status. Then, similar to aim 1, we will use ANCOVA to test the relationship between SMS and prescription opioid use; the variable selection process will be based on the significance of variables and the adjusted *R*^2^, while the medical marijuana use status will not be included in the list of variables for the selection. This will tell us if prescription opioid use affects driving performance regardless of medical marijuana use status. Additionally, if the best-fitted model contains different covariates from the best model for medical marijuana use seen in aim 1, we will also perform a model to adjust the same covariates as in aim 1 to compare the results for prescription opioid use and for medical marijuana use. This will allow us to see which one alone has a bigger effect on driving performance when adjusting for the corresponding and the same covariates.

#### Hypothesis 2b

Then we will check the combined effect of medical marijuana and prescription opioid on driving performance (SMS) using ANCOVA. The best fitted model we find in aim 1 will be performed in the subgroup of medical marijuana users and non–marijuana users, respectively. Thus, we will have a clear picture of the driving performance in the 4 groups: medical marijuana and prescription opioid, medical marijuana without prescription opioid, prescription opioid without medical marijuana, and no medical marijuana no prescription opioid. Thus, the combined effect can be determined.

### Alternative Design Plan

Limitations may be related to the distribution of the dependent variable, as ANCOVA has strict assumptions. An alternative plan would be to use generalized linear mixed models, which can handle non-Gaussian data, correlated data, unbalanced data, and address missing data while still maintaining power. Possible mitigation approaches include creating a dichotomous variable of high versus low for outcomes and using logistic generalized linear mixed model regression or other functional form as dictated by the distribution of the dependent and independent variables.

### Missing Data

Regarding missing data, we will also perform sensitivity analyses to assess the impact of missing data. Further, we will perform appropriate secondary analyses to adjust for variables that may be related to systematic missingness.

### Qualitative Aim Analysis

We will use content analysis, an analytic approach that allows qualitative data to be systematically gathered, described, and analyzed, with an emphasis on identifying patterned meaning across the data to identify key concepts, themes, subthemes [44]. A deductive approach to coding will be used, guided by the 3 theoretical frameworks described above. Both theoretical and investigator triangulation will be used to ensure the qualitative trustworthiness of the data. Using a mixed methods approach we will integrate both qualitative and quantitative data. We will use a quantitative plus qualitative structure, which is the simultaneous collection and analysis of quantitative and qualitative data, giving equal weight to both types of data. The function of the mixed methods approach is for development, with the long-term goal of developing effective intervention procedures to optimize risk screening, guidelines, and driver safety for adults aged 50 years and older regarding long-term medical marijuana and prescription opioid use. We will use a merge process to converge the two datasets, integrating quantitative and qualitative data by connecting the datasets to build on each other using NVivo (QSR International Pty Ltd).

## Results

Recruitment for the study began in May 2025, with the first participant enrolled the same month. As of the time of manuscript submission, a total of 30 participants have been enrolled. Recruitment is anticipated to be completed by 2029. The complete results and data from the study are expected to be published by 2030. Informed consent is obtained prior to beginning any study procedures. Participation is completely voluntary. Participant privacy and data confidentiality are protected with a number of measures, including a private interview setting for all study procedures, a HIPAA-compliant data storage platform (REDCap) and text message delivery application (Mosio), and coding of all data such that it cannot be associated with any individual. Participants will receive up to $245 if all assessments are completed as directed in the study protocol.

## Discussion

### Expected Findings

Our work will (1) focus on medical marijuana use with a sample who has used daily or most days for 12 months or longer, (2) confirm medical marijuana use based on date of card qualification and purchase history—all products used in the previous year will be downloaded from the OMMU to quantify use patterns over the course of the past year, (3) quantify medical marijuana use for 7 days before the on-road driving performance task using twice-daily ecological momentary assessment for all MMG participants, (4) measure prescription opioid using urinalysis on the day of the drive, and (5) measure driving performance using an open-road course under the observation of a CDRS or DRT trained to evaluate impaired driving in a test vehicle with the needed evaluator controls (eg, eye check mirrors and auxiliary brake) to maneuver the car and prevent any adverse occurrences, including near misses or crashes, if any dangerous situations arise. Older adults are more likely to be involved in crashes at intersections or during merges, and these crashes are inherently more fatal [45,46]. The challenging, fast-paced environment presented by intersections and merges puts extra demand on cognitive, sensory, and motor domains critical for driving, and slower response time, inattention, and difficulty with executive functions may lead to more driving errors, potential crashes, or fatal crashes [44]. Therefore, measurement using a standardized open-road course with a clinical CDRS or DRT in the passenger seat of the vehicle will ensure optimal safety of the driver and other road users. Specifically, we will provide state-of-the-art technology (a 2019 Toyota Corolla and a 2020 Toyota Camry, both equipped with instrumentation) for high-impact, low-risk, rigorous measurement of driving to assess users under the influence of medical marijuana and the combined effect of prescription opioid use. As medical marijuana use grows, prescription opioid use continues, and the US population ages, this study provides needed data to develop risk screening, recommendation guidelines, and relevant interventions for this population.

### Strengths and Limitations

As with any trial, our proposed study faces design and feasibility challenges. First, recruiting and retaining 250 older adults within the scope of our eligibility criteria may be challenging due to the complexity of the study, given that older adults are less inclined to engage with technology such as ecological momentary assessments or may feel uncomfortable performing an activity such as driving while being evaluated. However, we have experience with this population and an excellent recruitment infrastructure, which increases confidence in the study and provides the needed guidance with technology. Further, we have built in support for a dedicated research assistant to allow for the time and resources to onboard, train, and problem-solve throughout the assessment period. With guidance from our bioethicist, we have a predetermined protocol for anomalous findings suggesting impairment, which will be provided to participants upfront to ease concerns regarding potential loss of driving privileges. The goal of this study is to assess effects, not make clinical judgments; therefore, our on-road outcome is the SMS, and not the global rating scale of pass or fail outcomes. If we have concern about the on-road performance of a participant, we will report back to the referring physician, who may choose to counsel their patient accordingly. Given our experience with this population, we do not anticipate any difficulties that cannot be managed. Second, is the issue of generalizability. Since we will be using convenience sampling at our partner sites with stringent eligibility criteria, our sample may not be representative of all adults aged 50 years and older. However, since our goal is to determine the effect of an exposure (medical marijuana) on an outcome, representativeness is not necessary. Finally, it is possible that we will need to consider alternative factors that may influence our outcome, such as sex as a moderator due to dosage or titration schedules. We have considered this and are confident that we have the statistical expertise to address this issue as needed.

### Conclusions and Future Directions

We know a large percentage of adults aged 50 years and older who are using medical marijuana report use daily or most days of the week. A substantial percentage of adults aged 50 years and older are also using medical marijuana combined with prescription opioids. Previous data indicate that medical marijuana use patterns are associated with driving performance outcomes, particularly in the domains of divided attention and executive functioning. However, lack of vehicle feedback and kinesthetic-only (paper-and-pen) assessment of divided attention and executive function are insufficient. Due to the complexity of driving, evaluation by a CDRS or DRT is needed in order to assess the effectiveness of driving performance under real-world conditions. Data from the first ROAD ACE (1R21DA048067) indicate that our prospective study design and follow-up time period of 3 months is insufficient to identify the full extent of the role medical marijuana use plays in driving performance outcomes. Further, prescription opioid use needs assessment. This current proposal’s rigorous measurement of medical marijuana use combined with an open-road driving evaluation based on a valid and reliable measure, combined with our team’s expertise, will provide high-impact data that transforms the field by identifying risks that could be screened for and identifying needed intervention targets for self-regulation of use to support driving performance in this population.

## Data Availability

The datasets generated or analyzed during this study are not publicly available due the timing of data submission to our identified repository, but are available from the corresponding author on reasonable request.

## Appendix

Multimedia Appendix 1 Road Course and Scoring Documentation.

## Abbreviations

ANCOVA: analysis of covariance
AS1: assessment session 1
AS2: assessment session 2
AS3: assessment session 3
CDRS: certified driver rehabilitation specialist
CONSORT: Consolidated Standards of Reporting Trials
DRT: driver rehabilitation therapist
HIPAA: Health Insurance Portability and Accountability Act
MMG: medical marijuana group
MMSE-2: BV: Mini-Mental State Examination–Second Edition Brief Version
MMTC: Medical Marijuana Treatment Clinics of Florida
MoCA: Montreal Cognitive Assessment
NMG: no marijuana group
OMMU: Office of Medical Marijuana Use
REDCap: Research Electronic Data Capture
ROAD ACE: Reactions of Older Adults Driving After Cannabis Exposure
SMS: sum of maneuvers score
THC: tetrahydrocannabinol
THC-COOH: 11-nor-9-carboxy-tetrahydrocannabinol
UF: University of Florida

## References

[1] Bondallaz P, Favrat B, Chtioui H, Fornari E, Maeder P, Giroud C (2016). Cannabis and its effects on driving skills. Forensic Sci Int.

[2] Berning A, Compton R, Wochinger K (2015). 2013-14 national roadside study of alcohol and drug use by drivers. Department of Transportation. National Highway Traffic Safety Administration.

[3] Preuss UW, Huestis MA, Schneider M, Hermann D, Lutz B, Hasan A, Kambeitz J, Wong JWM, Hoch E (2021). Cannabis use and car crashes: a review. Front Psychiatry.

[4] Asbridge M, Hayden JA, Cartwright JL (2012). Acute cannabis consumption and motor vehicle collision risk: systematic review of observational studies and meta-analysis. BMJ.

[5] Brubacher JR, Chan H, Erdelyi S, Macdonald S, Asbridge M, Mann RE, Eppler J, Lund A, MacPherson A, Martz W, Schreiber WE, Brant R, Purssell RA (2019). Cannabis use as a risk factor for causing motor vehicle crashes: a prospective study. Addiction.

[6] Chihuri S, Li G (2019). Use of prescription opioids and initiation of fatal 2-vehicle crashes. JAMA Netw Open.

[7] Chihuri S, Li G (2017). Use of prescription opioids and motor vehicle crashes: a meta analysis. Accid Anal Prev.

[8] Sawyer P, Bodner EV, Ritchie CS, Allman RM (2006). Pain and pain medication use in community-dwelling older adults. Am J Geriatr Pharmacother.

[9] Reid MC, Eccleston C, Pillemer K (2015). Management of chronic pain in older adults. BMJ.

[10] Cameron-Burr KT, Conicella A, Neavyn MJ (2021). Opioid use and driving performance. J Med Toxicol.

[11] Guan Q, McCormack D, Juurlink DN, Bronskill SE, Wunsch H, Gomes T (2021). New opioid use and risk of emergency department visits related to motor vehicle collisions in Ontario, Canada. JAMA Netw Open.

[12] Maher RL, Hanlon J, Hajjar ER (2013). Clinical consequences of polypharmacy in elderly. Expert Opinion on Drug Safety.

[13] Borgelt LM, Franson KL, Nussbaum AM, Wang GS (2013). The pharmacologic and clinical effects of medical cannabis. Pharmacotherapy.

[14] Borgelt LM, Franson KL, Nussbaum AM, Wang GS (2013). The pharmacologic and clinical effects of medical cannabis. Pharmacotherapy.

[15] Broyd SJ, van Hell HH, Beale C, Yücel M, Solowij N (2016). Acute and chronic effects of cannabinoids on human cognition-a systematic review. Biol Psychiatry.

[16] Williams RH, Erickson T (2000). Emergency diagnosis of opioid intoxication. Lab Med.

[17] Fareed A, Stout S, Casarella J, Vayalapalli S, Cox J, Drexler K (2011). Illicit opioid intoxication: diagnosis and treatment. Subst Abuse.

[18] Clouston SAP, Brewster P, Kuh D, Richards M, Cooper R, Hardy R, Rubin MS, Hofer SM (2013). The dynamic relationship between physical function and cognition in longitudinal aging cohorts. Epidemiol Rev.

[19] Glisky EL (2007). Changes in cognitive function in human aging. Frontiers in Neuroscience.

[20] Ahamed A, Kullmann K, Frasso R, Goldstein J (2020). Trends in cannabis use among older adults in the United States, 2015-2018. JAMA Internal Medicine.

[21] Han BH, Le A, Funk-White M, Palamar JJ (2021). Cannabis and prescription drug use among older adults with functional impairment. Am J Prev Med.

[22] Paulozzi LJ, Strickler GK, Kreiner PW, Koris CM, Centers for Disease ControlPrevention (CDC) (2015). Controlled substance prescribing patterns--prescription behavior surveillance system, eight states, 2013. MMWR Surveill Summ.

[23] Cieza A, Brockow T, Ewert T, Amman E, Kollerits B, Chatterji S, Ustün T B, Stucki G (2002). Linking health-status measurements to the international classification of functioning, disability and health. J Rehabil Med.

[24] Gabrys RL, Porath AJ (2019). Clearing the Smoke on Cannabis: Regular Use and Cognitive Functioning.

[25] Kroon E, Kuhns L, Cousijn J (2021). The short-term and long-term effects of cannabis on cognition: recent advances in the field. Curr Opin Psychol.

[26] Meier MH, Caspi A, Ambler A, Harrington H, Houts R, Keefe RSE, McDonald K, Ward A, Poulton R, Moffitt TE (2012). Persistent cannabis users show neuropsychological decline from childhood to midlife. Proc Natl Acad Sci U S A.

[27] Bélanger A, Gagnon S, Stinchcombe A (2015). Crash avoidance in response to challenging driving events: the roles of age, serialization, and driving simulator platform. Accid Anal Prev.

[28] Baldock MRJ, Mathias JL, McLean J, Berndt A (2006). Self-regulation of driving and older drivers' functional abilities. Clinical Gerontologist.

[29] Dickerson A, Molnar L, Eby D, Adler G, Bédard M, Berg-Weger M, Classen S, Foley D, Horowitz A, Kerschner H, Page O, Silverstein NM, Staplin L, Trujillo L (2007). Transportation and aging: a research agenda for advancing safe mobility. Gerontologist.

[30] Eby DW, Molnar LJ (2009). Older adult safety and mobility. Public Works Management & Policy.

[31] (2016). Distribution of licensed drivers - 2015 by sex and percentage in each age group and relation to population. Florida Highway Administration.

[32] Allen HK, Beck KH, Zanjani F (2019). Driving concerns among older adults: associations with driving skill, behaviors, and experiences. Traffic Inj Prev.

[33] Toups R, Chirles TJ, Ehsani JP, Michael JP, Bernstein JPK, Calamia M, Parsons TD, Carr DB, Keller JN (2022). Driving performance in older adults: current measures, findings, and implications for roadway safety. Innov Aging.

[34] Justiss MD, Mann WC, Stav W, Velozo C (2006). Development of a behind-the-wheel driving performance assessment for older adults. Topics in Geriatric Rehabilitation.

[35] Stav WB, Justiss MD, McCarthy DP, Mann WC, Lanford DN (2008). Predictability of clinical assessments for driving performance. J Safety Res.

[36] Kazdin A (2022). Reserach Design in Clincial Psychology.

[37] Lee Y, Lee S, Chiu E (2022). Practice effect and test-retest reliability of the Mini-Mental State Examination-2 in people with dementia. BMC Geriatr.

[38] Nasreddine ZS, Phillips NA, Bédirian V, Charbonneau S, Whitehead V, Collin I, Cummings JL, Chertkow H (2005). The Montreal Cognitive Assessment, MoCA: a brief screening tool for mild cognitive impairment. J Am Geriatr Soc.

[39] Downey LA, Hayley AC, Porath-Waller AJ, Boorman M, Stough C (2016). The standardized field sobriety tests (SFST) and measures of cognitive functioning. Accid Anal Prev.

[40] Doroudgar S, Mae Chuang H, Bohnert K, Canedo J, Burrowes S, Perry PJ (2018). Effects of chronic marijuana use on driving performance. Traffic Inj Prev.

[41] Hennink M, Kaiser BN (2022). Sample sizes for saturation in qualitative research: a systematic review of empirical tests. Soc Sci Med.

[42] Integrated Data Repository Research Services?» Clinical and Translational Science Institute?.

[43] i2b2.

[44] Hsieh H, Shannon SE (2005). Three approaches to qualitative content analysis. Qual Health Res.

[45] Lombardi DA, Horrey WJ, Courtney TK (2017). Age-related differences in fatal intersection crashes in the United States. Accid Anal Prev.

[46] Yamani Y, Horrey WJ, Liang Y, Fisher DL (2016). Age-related differences in vehicle control and eye movement patterns at intersections: older and middle-aged drivers. PLoS One.

