# Supplementary material for: An Open-Road Driving Performance Task to Examine Long-Term Medical Marijuana Use and Prescription Opioid Positivity Among Adults Aged 50 Years and Older: Protocol for an Observational Trial
Source: JMIR Res Protoc. 2025 Nov 28;14:e77944. doi: 10.2196/77944 (PMC12701343; doi:10.2196/77944)
Supplement: Multimedia Appendix 1 [file resprot_v14i1e77944_app1.pdf]

STUDY00005560
FSU IRB Approved
13 November 2024

Zone	Location	Mileage	Controlled	Uncontrolled
Familiarization to vehicle	Oak Hammock			
Residential<br><br>Section distance 1.2 miles	Exit Gatehouse from Oak Hammock onto SW 25th PL → SW 40th PL	0..00	1	0
	LEFT on SW 40th PL		1	0
	SW 40th PL → SW 26th DR		0	1
	RIGHT on SW 26th DR		0	1
	SW 26th DR → SW 38th PL		0	0
	LEFT on SW 38th PL		1	0
	SW 38th Pl → SW 27th ST		0	0
	RIGHT on SW 27th ST		1	0
	SW 27th ST → SW 35th PL		0	0
	RIGHT on SW 35th PL		1	0
	SW 35th PL → SW 23rd ST		0	3
	ROUNDABOUT 1ST EXIT → SW 23rd ST	1.2	1	0
Suburban roadway<br><br>Section length 4.1 miles	SW 23rd ST → SW Williston RD		0	0
	LEFT on SW Williston RD		1	0
	SW Williston RD → S Main ST		1	6
	LEFT on S Main		0	1
	S Main St. → SW 16th AVE		0	5
	LEFT on SW 16th AVE		1	0
	SW 16th AVE → SW 6th ST		0	0
	RIGHT on SW 6th ST		1	0
	SW 6th ST → SW 4th AVE		1	2
	ROUNDABOUT 1ST EXIT → SW 4th AVE	5.3	1	0
Downtown – congested<br><br>Section length 1.8 miles	SW 4th AVE → S Main ST		1	3
	LEFT on S Main ST		1	0
	S Main ST → SW 2nd AVE		0	2
	LEFT on SW 2nd AVE		1	0
	SW 2nd AVE → SW 6th ST		0	5
	ROUNDABOUT 3rd EXIT → SW 6th ST		1	0
	SW 6th ST → SW Depot AVE		1	2
	ROUNDABOUT 1ST EXIT → SW Depot Ave		1	0
	SW Depot Ave → SW 9th RD		0	2
	ROUNDABOUT 2nd EXIT → SW 9th RD		1	0
	SW 9th RD → SW 13th ST		0	0
	LEFT on SW 13th ST	7.1	1	0
Suburban roadway<br><br>Section length 3.8 miles	SW 13th ST → SW Williston RD		2	7
	RIGHT on SW Williston RD		1	0
	SW Williston RD → SW 29th DR		2	7
	LEFT on SW 29th DR		0	1
	SW 29th DR → Smart House	10.9	0	1
	TOTAL	10.9	25	49

STUDY00005560
FSU IRB Approved
13 November 2024

Legend

- Controlled intersection has traffic control in direction of travel by either 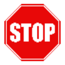

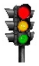 or 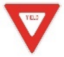
- Uncontrolled intersection no traffic control in direction of travel. Side roads may have stop signs.
- Grey: Familiarization drive 2 lane roads 15 mph 3-5 minutes
- Green: Residential speeds 25 – 30 mph, 2 lane roadways
- Blue: Suburban speeds 35 – 45 mph, 2 – 4 lane divided roadways
- Orange: Downtown – Congested 2 lane roads speeds 25 – 35 mph, higher level of pedestrians and cyclists

Study Route – does not include familiarization drive

- Total Length 10.9 miles
- Time ≈ 25 minutes
- 25 - Controlled intersections
- 49 - Uncontrolled intersections
- 9 - Left turns
- 5 - Right turns
- 7 - Roundabouts
- 2 – Merges due to lanes ending
- 11 - Required lane changes
- Intersections connected via straight roadways.
- Speed limits vary between 25 – 45 mph on two-lane, four- lane and divided highways.

Section A: Orientation to vehicle

Participant #:		Date/Start Time
Research Driver Rehabilitation Therapist:		Vehicle used 2019 Camry 2020 Corolla
Weather: Sunny Cloudy Light rain	Temp:	License check - Valid

Section B: Orientation to vehicle

☐Driving seat adjustment ☐Steering wheel adjustment ☐Ignition ☐Mirror adjustment ☐Instrument displays ☐Accelerator and Brake ☐Turn indicators ☐Gearshift ☐Instructor brake.

Section C: Familiarization Drive (Duration: 3 minutes)

General instruction to all clients at the start of Familiarization drive: **If there has been no turn instruction, continue straight. Please obey traffic rules.”** Ask **“Are you familiar with roundabouts?”** If no, explain right of way and signaling rules and the concept of exits.

Speed limit	*[Trigger]* – Verbal Instructions – Task/location/maneuver	VS	SR	LM	Si	VP	Yi	GA	Adj St	OK	Score	Scored maneuver/Comments
**15 mph**	*[Back out of driveway to face SW 53rd Ln]*<br>**Back out of the driveway so that you face that way.**<br>(Gesture towards SW 53rd Ln)											
	[After client stops at corner of SW 53rd Ln]<br>**Make a left turn.**											Left turn
	(Straight on SW 53rd Ln)											Straight drive
	[Immediately after passing parking lot on right]<br>**Turn right at the next side street.**<br>(Right turn onto SW 50th Blvd)											Right turn
	(Straight drive)											Straight drive/Ped X
	[After passing N. Service Dr.]<br>**Please park in a space on the right side of the roadway.**<br>(Client should identify a parking place and once parked shift to Park)											Parking/right turn
	**When safe, back out and continue in the previous direction.**<br>(Continues down SW 50th Blvd)											Straight drive
	[Immediately after shifting to drive]<br>**Turn left at the next intersection.**<br>(Left turn at 4-way stop onto SW 25th Blvd.)											Left turn
	[Driving on SW 25th Blvd]<br>**Continue to the guard gate.**<br>(Curving 2 lane road with center line)											Straight drive
	[When approaching guard house]<br>**Pull slowly up to the gate and it will open automatically.**<br>(Client should proceed through gate)											Straight drive
	(Client should make left turn at stop sign onto SW 25th PL, no cue given as this is the only option)											Left turn

Note: Driving errors made during the Familiarization Drive are not included in the final scoring.

 VS = Visual Scan SR= Speed Regulation LM= Lane Maintenance Si= Signaling VP= Vehicle Position Yi= Yielding GA= Gap Acceptance Adj St= Adjustment to Stimuli

STUDY00005560
FSU IRB Approved
13 November 2024

Section D: Driving Route: Duration 25 minutes (as per Google maps)

General Instruction **"Follow the instructions that I give you. Only proceed when it is safe to do so and obey all road rules. If there is no instruction to turn, continue straight on. Any questions?"**

Speed limit	*[Trigger]* – Verbal Instructions – (Task/location/maneuver)	VS	SR	LM	Si	VP	Yi	GA	Adj St	OK	Score	Scored maneuver/Comments
**25 mph**	*[Immediately after left turn onto SW 25th PL]*<br>**At the traffic light continue straight.**<br>(Crossing SW Williston Rd. at traffic light continuing SW 25th PL)											Straight drive
	[Immediately after crossing Williston Rd.]<br>**At the next intersection turn left**<br>(Straight driving to stop sign T intersection one-way stop)											Straight drive
	(left turn onto SW 40th PL, continues straight)											Left turn UP
	[Immediately after completing turn.]<br>**At the second street turn right.**<br>(Right turn onto SW 26th DR)											Straight drive
	(Right turn onto SW 26th DR continues straight)											Right turn
	[Immediately after completing turn.]<br>**At the next intersection turn left**<br>(Straight on SW 26th DR to stop sign)											Straight drive
	(Left turn onto SW 38th PL. T intersection one-way stop)											Left turn UP
	[Immediately after completing turn.]<br>**At the next intersection turn right.**<br>(Straight on SW 38th PL to stop sign)											Straight drive
	(Right turn onto SW 27th ST. Two-way stop.)											Right turn UP
**30 mph**	*[Immediately after completing turn.]*<br>**Speed limit is now 30 mph. At the end of the street turn right.**<br>(Straight on SW 27th ST to stop sign)											Straight drive
	(Right turn onto SW 35th PL. T intersection one-way stop)											Right turn UP
	[Immediately after completing turn.]<br>**At the roundabout take the first exit.**<br>(Continues straight on SW 35th PL. until roundabout)											Straight drive
	(Takes first exit from roundabout onto SW 23rd ST)											Roundabout

 VS = Visual Scan SR= Speed Regulation LM= Lane Maintenance Si= Signaling VP= Vehicle Position Yi= Yielding GA= Gap Acceptance Adj St= Adjustment to Stimuli

Speed limit	*[Trigger]* – Verbal Instructions – (Task/location/maneuver)	VS	SR	LM	Si	VP	Yi	GA	Adj St	OK	Score	Scored maneuver/Comments
40 mph	*[Immediately after exiting the roundabout.]*<br>**At the traffic light turn left.**<br>(Straight on SW 23h ST, Lane change to left turn lane)											Straight drive/lane change
	(Left turn onto SW Williston RD. Traffic light 4 lane divided roadway.)											Left turn UP
45 mph	*[Immediately after completing turn if client is in the left lane.]*<br>**The speed limit is 45 mph. When it is safe make a lane change to the right.**<br>(Lane change continues straight on SW Williston RD)											Straight drive/lane change
45 – 35 – 45 mph	[*Immediately after passing End School Zone sign*]<br>**Go straight through the next traffic light, after that we will be making a left turn.**<br>(Straight on SW Williston RD, should make lane change to left in preparation for turn. Speed limit drops prior to traffic light then goes back up.)											Straight drive/lane change
45 mph	[After crossing 441 @ traffic light and passing U-Haul]<br>**See the green sign on the left? After the green sign comes a left turn lane, move to the turn lane, and make a left onto Main ST.**<br>(Continues straight on SW Williston RD, should make lane change to left in preparation for turn and lane change into left turn lane)											Straight drive/lane change
	(Left turn onto S Main ST. Unprotected turn across 4 lane divided roadway.)											Left turn UP
	[Immediately after completing turn.]<br>**When it is safe make a lane change to the right.**<br>(Continues straight on S Main ST.)											Straight drive/lane change
	(Right lane ends sign, merge left, no cues.)											High speed merge
	[*Immediately after client completes merge]*<br>**At the traffic light turn left.**<br>(Straight on S Main ST, lane change to turn lane)											Straight drive/lane change
	(Left turn onto SW 16th AVE. 4-lane divided road)											Left turn
35 mph	*[Immediately after completing turn.]*<br>**At the next traffic light turn right.**<br>(On SW 16th AV must make lane change to right, if not already in right lane)											Straight drive/lane change

 VS = Visual Scan SR= Speed Regulation LM= Lane Maintenance Si= Signaling VP= Vehicle Position Yi= Yielding GA= Gap Acceptance Adj St= Adjustment to Stimuli

STUDY00005560
FSU IRB Approved
13 November 2024

**Speed limit**	*[Trigger]* **– Verbal Instructions –** (Task/location/maneuver)	**VS**	**SR**	**LM**	**Si**	**VP**	**Yi**	**GA**	**Adj St**	**OK**	**Score**	**Scored maneuver/Comments**
	(Right turn onto SW 6th ST, 4-lane road should turn into right lane)											Right turn
**35 mph**	*[Immediately after completing turn.]*<br>**At the second roundabout take the first exit.**<br>(Right lane ends sign, client needs to merge left no cues.)											Straight drive/merge left
	(Continues through 1st roundabout no cues)											Roundabout
**30 mph**	(Continues straight on SW 6th ST to 2nd roundabout speed limit drops, multiple ped X)											Straight drive
	(Takes the first exit from the roundabout onto SW 4th AVE)											Roundabout

 VS = Visual Scan SR= Speed Regulation LM= Lane Maintenance Si= Signaling VP= Vehicle Position Yi= Yielding GA= Gap Acceptance Adj St= Adjustment to Stimuli

STUDY00005560
FSU IRB Approved
13 November 2024

Speed limit	*[Trigger]* – Verbal Instructions – (Task/location/maneuver)	VS	SR	LM	Si	VP	Yi	GA	Adj St	OK	Score	Scored maneuver/Comments
25 mph	*[Immediately after exiting the roundabout.]*<br>**At the traffic light turn left.**<br>(Straight on SW 4th AVE)											Straight drive
	(Stop at all-way stop sign mid-block no cues)											Stop sign/all way
	(Continue straight, lane shifts to right, lane change to left turn lane approaching traffic light)											Straight drive/lane change
	(Left turn onto S Main ST.)											Left turn
30 mph	*[Immediately after completing turn.]*<br>**At the next traffic light turn left.**<br>(Straight on S Main ST. must make lane change into turn lane at traffic light)											Straight drive/lane change
	(Left turn onto SW 2nd AVE)											Left turn
	[Immediately after completing turn.]<br>**At the next roundabout take the third exit.**<br>(Continues straight on SW 2nd AVE to roundabout)											Straight drive
	(Takes the third exit from the roundabout onto SW 6th ST)											Roundabout
	[Immediately after exiting roundabout.]<br>**At the second roundabout take the first exit.**<br>(Straight on SW 6th ST to second roundabout)											Straight drive
	(Continues through first roundabout no cues)											Roundabout
	(Second roundabout takes first exit onto SW Depot AVE											Roundabout
25 mph	*[Immediately after exiting roundabout.]*<br>**At the next roundabout take the second exit.**<br>(Straight on SW Depot Ave. to next roundabout)											Straight drive
	(Exit roundabout at second exit onto SW 9th RD)											Roundabout
	[Immediately after exiting roundabout.]<br>**At the traffic light make a left**<br>(Straight on SW 9th Rd)											Straight drive
	(Left turn onto SW 13 ST)											Left turn

 VS = Visual Scan SR= Speed Regulation LM= Lane Maintenance Si= Signaling VP= Vehicle Position Yi= Yielding GA= Gap Acceptance Adj St= Adjustment to Stimuli

STUDY00005560
FSU IRB Approved
13 November 2024

Speed limit	*[Trigger]* – Verbal Instructions – (Task/location/maneuver)	VS	SR	LM	Si	VP	Yi	GA	Adj St	OK	Score	Scored maneuver/Comments
35 -45 mph	*[Immediately after completing turn]*<br>**At the third light turn right**<br>(Straight on SW 13th ST 4-lane divided road, speed limit increases, will need to make a lane to the right eventually)											Straight drive/lane change
	(Right turn at traffic light onto SW Williston RD)											Right turn
45 mph	*[After the light at Oak Hammock SW 25th PL]*<br>**We will be making a left turn at the end of the median.**<br>(Straight on SW Williston should make lane change to left turn lane)											Straight drive/lane change(s)
	(Left turn onto SW 29th Dr.)											Left turn UP
25 mph	*[Immediately after completing turn.]*<br>**Continue straight to the gate, pull up slowly and proceed when open.**<br>(Straight on SW 29th Dr., direct client to Smart House to park)											Straight drive
	Totals for errors for each driving behavior											
	Total score											Additional comments
	• Total number of scored maneuvers = 49<br>• Optimal score (no driving errors) = 147<br>• Total number of potential scored maneuvers in each error category:<br>VS = 49<br>SR = 49<br>LM = 49<br>Si = 35<br>VP = 49<br>Yi = 36<br>GA = 20<br>Adj St = 49<br><br>***Note**: More than one occurrence of an error may be possible in each section/maneuver. For example, within the same straight drive the client could make more than one speed regulation or lane positioning error. Each occurrence is denoted by a tick mark so the total number of errors in each behavior category can be calculated.											

 VS = Visual Scan SR= Speed Regulation LM= Lane Maintenance Si= Signaling VP= Vehicle Position Yi= Yielding GA= Gap Acceptance Adj St= Adjustment to Stimuli

STUDY00005560
FSU IRB Approved
13 November 2024

DEFINITIONS AND SCORING KEY

- **VS=** Visual scanning **-** Demonstrating visual scanning of driving environment. Examples of errors: Not checking blind spot or mirrors during turns and lane changes, not looking left/right before proceeding through intersection.
- **SR**= Speed regulation - Reflects ability to follow and maintain speed limits and having adequate control of acceleration and braking features of the vehicle. Examples of errors: not coming to a complete stop at stop sign, traveling too slow/fast, inadequate merging speed, abrupt or inappropriate braking or acceleration.
- **LM**= Lane maintenance - Refers to the lateral (side-to-side) positioning of the vehicle during driving maneuvers (turns, straight driving, lane changes, etc.) and while stopped. This reflects the ability to maintain steering control.
- **Si**= Signaling - Reflects the proper use of turn signals. Examples of errors: leaving the turn signal on, not using the turn signal when turning, using the turn signal inappropriately (wrong signal for given turn, signaling too short until maneuver).
- **VP**= Vehicle positioning - Vehicle position refers to the position of the vehicle forward and backward (anterior-posterior) in relation to other vehicles and/or objects and pavement markings. This category captures following distance during forward movement and vehicle spacing during lane changes and merges. Examples of errors: traveling too closely (tailgating), inadequate space cushion during merge or lane change, stopping across a crosswalk or too far back from either pavement markings or other vehicles.
- **Yi**= Yielding - Giving right-of-way when appropriate. Yielding refers to the ability to recognize common rules of road safety. Yielding is assessed at 4-way or 2-way stop intersections (when other vehicles are present), right turns on red, and merges.
- **GA**= Gap acceptance - Choosing an appropriately safe time and/or spacing distance to cross in front of oncoming traffic (unprotected left turn). Errors in gap acceptance are based on evaluator judgment given the speed of oncoming traffic and number of lanes to be crossed.
- **Adj St**= Adjustment -to -stimuli - Ability to appropriately respond to driving situations. This captures ability to adjust appropriately to changing road sign information, other vehicle movements, pedestrian movements, and ability to recognize potential hazards. Examples of errors: not adjusting speed for posted limits, not following proper directions given by evaluator, choosing improper lane from posted signage, improper response to traffic, pedestrian, or cyclist movement.

Note: Grey shading in the Table indicates where a specific type of error cannot occur or be scored.
Note: The *trigger* prompts the research driver rehabilitation specialist to provide the following verbal instruction at a specific point in the route.

Intervention codes - Tally marks are occurrences **V**= verbal cue **P**= physical assist Check mark in OK= No errors or Intervention needed

CHART COLOR CODE

- Grey: Familiarization drive 2 lane roads 15 mph 3-5 minutes
- Green: Residential speeds 25 – 30 mph, 2 lane roadways
- Blue: Suburban speeds 35 – 45 mph, 2 – 4 lane divided roadways
- Orange: Downtown – Congested 2 lane roads speeds 25 – 35 mph, higher level of pedestrians and cyclists

SCORING

- 3 = no errors
- 2= any error in any driving behavior for the given maneuver
- 1= the evaluator must use verbal cues or repeat instructions (not hearing-related) to modify or change driving behavior
- 0= physical intervention (such as grabbing the steering wheel, using the auxiliary brake) is required.
